# Indices of Systemic Inflammation Are Elevated in Women With Evidence of Metabolic Syndrome After Anterior Cruciate Ligament Reconstruction

**DOI:** 10.1002/ars2.70091

**Published:** 2026-09-22

**Authors:** Marina Abdelshahid, Sonu Bae, Hayden Price, Hawraa Al Janabi, Catherine Rock, Anthony Mantor, Nicholas Apseloff, Christopher C. Kaeding, Robert A. Magnussen, David C. Flanigan, Tyler Barker

**Affiliations:** ^1^ Sports Medicine Research Institute, College of Medicine The Ohio State University Wexner Medical Center Columbus Ohio U.S.A.; ^2^ The Ohio State University College of Medicine Columbus Ohio U.S.A.; ^3^ Department of Orthopaedic Surgery College of Medicine The Ohio State University Wexner Medical Center Columbus Ohio U.S.A.

## Abstract

**Purpose:**

To investigate the association between metabolic syndrome (MetSy) and systemic indices of the immune system and inflammation after anterior cruciate ligament reconstruction (ACLR).

**Methods:**

This retrospective study included adult patients (≥18 years) who underwent ACLR at a single academic institution between 2009 and 2022. Patients with documented complete blood cell count ≥90 days postoperatively and complete MetSy component data were included. From complete blood cell data, the pan‐immune‐inflammation value (PIV), systemic inflammation response index (SIRI), systemic immune‐inflammatory index (SII), neutrophil‐to‐lymphocyte ratio (NLR), platelet‐to‐lymphocyte ratio, and monocyte‐to‐lymphocyte ratio were calculated. Evidence of MetSy was defined by ≥3 of the following: body mass index ≥30, triglycerides ≥150 mg/dL, high‐density lipoprotein < 40 mg/dL (men) or <50 mg/dL (women), estimated fasting glucose ≥ 100 mg/dL, and systolic blood pressure ≥130 mmHg or diastolic blood pressure ≥85 mmHg. Patients (n = 130) were grouped based on MetSy status at ACLR: MetSy or non‐MetSy.

**Results:**

A total of 130 patients were included: 50 MetSy and 80 non‐MetSy. The NLR, monocyte‐to‐lymphocyte ratio, platelet‐to‐lymphocyte ratio, SIRI, SII, and PIV did not differ significantly between MetSy groups. However, NLR, platelet‐to‐lymphocyte ratio, SII, and PIV were significantly elevated (*P* < .01, *P* = .04, *P* < .001, and *P* < .01, respectively) in women compared with men with MetSy. Patients with higher body mass index and fasting glucose displayed elevated PIV (*P* = .01 and *P* = .03), whereas low high‐density lipoprotein was associated with an elevation in NLR, SIRI, SII, and PIV (*P* = .04, *P* = .02, *P* = .04, and *P* = .02, respectively). The monocyte‐to‐lymphocyte ratio, SIRI, SII, and PIV were elevated with postoperative complications (*P* = .03, *P* < .01, *P* = .03, and *P* < .01, respectively).

**Conclusions:**

Indices of systemic inflammation after ACLR were elevated in women with evidence of MetSy. Patients with postoperative complications also exhibited an elevation in systemic indices of inflammation after ACLR.

**Level of Evidence:**

Level III, retrospective comparative study.

## Graphical abstract

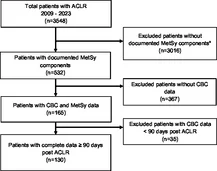

Anterior cruciate ligament (ACL) injury is one of the most common knee injuries. Women are 2 to 8 times more at risk of ACL injury and have lower patient‐reported outcomes in comparison to men in the early postoperative period.^1^, ^2^, ^3^, ^4^ Although the cause remains elusive, some studies suggest that hormonal, anatomical, and biochemical factors contribute to the increased risk of ACL injury.^5^, ^6^

Chronic systemic inflammation is associated with many chronic diseases, such as diabetes, rheumatoid arthritis, osteoarthritis, and poor postoperative recovery.^7^, ^8^ Age, obesity, and elevated sex hormones can promote a persistent inflammatory response.^9^, ^10^, ^11^ In clinical settings, C‐reactive protein, serum protein electrophoresis, and fibrinogen are inexpensive, readily used blood tests to assess inflammation.^8^ Additionally, there is a growing interest in the systemic immune and inflammatory indices, which are derived from a readily available complete blood cell (CBC) count.^12^, ^13^, ^14^ These indices include pan‐immune‐inflammation value (PIV), systemic inflammation response index (SIRI), systemic immune‐inflammatory index (SII), neutrophil‐to‐lymphocyte ratio (NLR), platelet‐to‐lymphocyte ratio (PLR), and monocyte‐to‐lymphocyte ratio (MLR). These indices have been used as a prognostic tool for many conditions, such as acute ischemic stroke, colorectal cancer, ovarian cancer, and cardiovascular disease.^15^, ^16^, ^17^, ^18^

Metabolic syndrome (MetSy) is a cluster of cardiovascular risk factors. There are multiple definitions for establishing MetSy criteria. The most widely used is the National Cholesterol Education Program Adult Treatment Panel III definition, which requires any 3 of the following 5 criteria to diagnose: hyperglycemia, obesity, low high‐density lipoprotein (HDL), dyslipidemia (high triglycerides), and hypertension.^19^, ^20^ The rising global prevalence of MetSy underscores the growing interest in investigating this complex, multifactorial condition. Notably, MetSy is associated with an increased risk of surgical complications, including surgical site infections, cardiovascular complications, and hospital readmissions across cardiac, emergency, gastrointestinal, and orthopaedic patients.^21^ Additionally, previous research suggests that MetSy is associated with chronic, low‐grade systemic inflammation, including inflammatory indices such as SII.^3^, ^10^, ^11^, ^19^ However, the potential impact of MetSy on systemic inflammation after ACL reconstruction (ACLR) is not well understood.

The purpose of this study was to investigate the association between MetSy and systemic indices of the immune system and inflammation after ACLR. We hypothesized that systemic indices of the immune system and inflammation are increased with MetSy after ACLR. Patients were grouped based on MetSy status, and study inclusion required the presence of documented MetSy components and CBC data obtained ≥90 days after ACLR.

## METHODS

This retrospective study included adult patients (≥18 years) who underwent arthroscopic ACLR for an ACL tear between August 2009 and October 2022 at The Ohio State University Wexner Medical Center. The last date of review and extraction from the electronic medical records was May 15, 2023. All data were extracted from the electronic medical records and aligned to the date of ACLR. This study complies with the Declaration of Helsinki and was approved by the governing Institutional Review Board (#2023H0283).

### Subjects

Patients who underwent ACLR were initially identified using the Current Procedural Terminology code 29888. Patients were subsequently screened for basic patient demographics, blood pressure (±30 days of ACLR), blood lipids, and hemoglobin A1c necessary for assessing MetSy. Patients with any missing components used to assess MetSy were excluded, whereas those with all documented components were initially included in this study. Patients were then screened for available CBC with differential data obtained ≥90 days after ACLR.

The final analysis included 130 patients who underwent ACLR with complete MetSy components and CBC data obtained ≥90 days after ACLR. Patients were subsequently separated into groups based on MetSy status: (1) MetSy or (2) non‐MetSy.

### Assessment of MetSy and Its Components

MetSy components were determined based on the National Cholesterol Education Program Adult Treatment Panel III, except for waist circumference.^20^ Waist circumference was not collected as a standard of care procedure and was subsequently substituted with body mass index (BMI). BMI is correlated with and previously used as a substitute for waist circumference in other MetSy studies.^22^ MetSy was defined as meeting any 3 of 5 criteria: (1) obesity (BMI ≥ 30), (2) dyslipidemia (triglycerides ≥150 mg/dL), (3) low HDL (men < 40 and women < 50 mg/dL), (4) hyperglycemia (estimated fasting glucose ≥ 100 mg/dL), and (5) hypertension (systolic blood pressure ≥ 130 mmHg or diastolic blood pressure ≥ 85 mmHg). Fasting glucose was estimated using the following equation:^23^

Estimated fasting glucose (mg/dL) = 28.7 × HbA1c – 46.7

### Blood Cell Counts and Systemic Indices of Inflammation

CBC counts were ordered for indications unrelated to ACLR routine care. The first available CBC obtained ≥90 days after ACLR was used in this study. The systemic immune system and inflammatory indices were calculated from the absolute counts from CBC and included the NLR (neutrophil/lymphocyte ratio), MLR (monocyte/lymphocyte ratio), PLR (platelet/lymphocyte ratio), SIRI (neutrophil × monocyte/lymphocyte), SII (platelet × neutrophil/lymphocyte), and PIV (neutrophil × platelet × monocyte/lymphocyte).

### Postoperative Outcomes

Data were collected on postoperative complications, ACLR‐related emergency department (ED) visits, subsequent surgeries (in the absence of reinjury), and reinjury events. Complications and other events were assessed with a minimum follow‐up of 8 months up to 4 years after ACLR. Subsequent surgeries included ipsilateral knee arthroscopy (with meniscectomy, synovectomy, chondroplasty, debridement, or lysis of adhesion), ACL revision, manipulation under anesthesia, and meniscus repair. Surgeries performed because of reinjury included ACL revision and arthroscopy (with meniscectomy, synovectomy, chondroplasty, or meniscus repair). Complications were identified and extracted from the electronic medical records. Defined complications included deep vein thrombosis, unexplained joint effusion, unexplained chronic pain, joint stiffness, quadriceps weakness, primary or progressive osteoarthritis, meniscus retear, limited range of motion, arthrofibrosis, wound infection, cartilage degeneration, cyclops lesions, joint laxity, and recurrent knee instability.

### Statistical Analysis

Data was assessed for normality using the Shapiro‐Wilk test and Kolmogorov‐Smirnov test before statistical analysis. Pearson's *χ* ^2^ test was performed to analyze the associations between categorical variables (MetSy vs non‐MetSy). Within each group, separate nonparametric Kruskal‐Wallis 1‐way ANOVA tests were conducted to evaluate whether each independent variable—age, BMI, and graft type—was associated with differences in each systemic index (NLR, MLR, PLR, SIRI, SII, and PIV). Wilcoxon rank‐sum tests were performed to assess differences in systemic indices across the MetSy criterion, sex, MetSy status, complication status, reinjury status, subsequent surgeries, and ED visits. Spearman correlation was performed to assess correlation between systemic indices and number of postoperative complications. Significance was set at *P* < .05. Bonferroni correction was not applied based on the exploratory nature of this study. All statistical analyses were performed with R (version 4.5.0, R Foundation for Statistical Computing, Vienna, Austria).

## RESULTS

### Subject Characteristics

The initial criteria identified 532 out of 3548 patients who underwent ACLR with documented MetSy component elements in their electronic medical records (Figure 1). Of those patients, 165 also possessed CBC data. Patients with a documented CBC less than 90 days after ACLR were excluded (n = 35). The final analysis was performed using 130 patients who underwent ACLR with documented CBC count ≥90 days after surgery. Patient sex, age, height, and race were not significantly different between MetSy status groups (Table 1). The graft type was significantly different between MetSy status groups (*P* < .01). Approximately 80% of the MetSy group and 20% of the non‐MetSy had a BMI considered obese or severely obese (*P* < .01).

**FIGURE 1:**
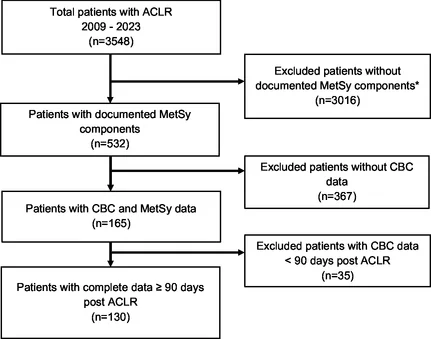
Subject inclusion flowchart. The initial inclusion criteria identified 532 out of 3548 patients who underwent ACLR with documented MetSy component elements in their electronic medical records. Of those patients, 165 also possessed CBC data. Thirty‐five patients with a documented CBC less than 90 days after ACLR were excluded. The final analysis included 130 patients who underwent arthroscopic ACLR with complete MetSy components and CBC data obtained ≥90 days after ACLR. Patients were subsequently separated into groups: (1) MetSy (n = 50) or (2) non‐MetSy (n = 80). *Blood pressure ± 30 days of ACLR, BMI, triglycerides, HDL, and estimated fasting glucose. (ACLR, anterior cruciate ligament reconstruction; BMI, body mass index; CBC, complete blood cell; HDL, high‐density lipoprotein; MetSy, metabolic syndrome.)

**TABLE 1:** Patient Characteristics

	**MetSy**	**Non‐MetSy**	***P*** **Value**
n (women/men)	50.0 (26/24)	80.0 (44/36)	.88∗
Age, yr	36.1 (21.4)	35.7 (13.6)	.58
Height, m	1.70 (0.15)	1.70 (0.15)	.86
Body mass, kg	102 (27.7)	78.0 (21.6)	<.01
BMI	32.7 (6.6)	25.7 (5.4)	<.01
BMI classifications			<.01
Underweight (≤18.5), n (%)	0	0	
Healthy weight (18.5‐24.9), n (%)	5.00 (10.0)	34.0 (42.5)	
Overweight (25.0‐29.9), n (%)	5.00 (10.0)	30.0 (37.5)	
Obese (30.0‐39.9), n (%)	32.0 (64.0)	15.0 (18.8)	
Severe obesity (≥40.0), n (%)	8.00 (16.0)	1.00 (1.20)	
Systolic blood pressure, mmHg	137 (12.6)	124 (18.1)	<.01
Diastolic blood pressure, mmHg	79.5 (9.70)	74.5 (10.7)	<.01
Triglycerides, mg/dL	176 (134)	98.0 (61.3)	<.01
HDL, mg/dL	41.5 (12.3)	53.5 (20.0)	<.01
LDL, mg/dL	110 (44.0)	103 (53.0)	.53
Estimated fasting glucose, mg/dL	111 (14.4)	102 (12.2)	<.01
CBC			
WBC, K/μL	7.62 (3.41)	6.79 (2.91)	.01
RBC, M/μL	4.81 (0.50)	4.59 (0.90)	.05
Platelet, K/μL	242 (96.0)	243 (94.0)	.72
Neutrophil, K/μL	4.64 (2.59)	3.79 (2.39)	.02
Lymphocyte, K/μL	2.14 (1.02)	1.90 (0.72)	.05
Monocyte, K/μL	0.57 (0.22)	0.50 (0.24)	.04
Race			.93
African American/Black, n (%)	8.00 (16.0)	10.0 (12.5)	
White, n (%)	35.0 (70.0)	56.0 (70.0)	
Asian, n (%)	2.00 (4.00)	6.00 (7.50)	
Other, n (%)	5.00 (10.0)	8.00 (10.0)	
Graft type			<.01
Allograft, n (%)	22.0 (44.0)	14.0 (17.5)	
BTB autograft, n (%)	4.00 (8.00)	4.00 (5.00)	
Hamstring autograft, n (%)	23.0 (46.0)	61.0 (76.3)	
Hybrid†, n (%)	1.00 (2.00)	1.00 (1.25)	

*Note*: Data are presented as median (IQR) and n (%) unless noted otherwise.

BMI, body mass index; BTB, bone–patellar tendon–bone; CBC, complete blood count; HDL, high‐density lipoprotein; LDL, low‐density lipoprotein; m, meters; MetSy, metabolic syndrome; RBC, red blood cell; WBC, white blood cell; yr, year.

∗ *P* value based on sex distribution (n) between groups.

† Hybrid graft is a combination of autograft and allograft.

### Blood Cell Counts

Red blood cell, platelet, and lymphocyte counts were not significantly different between MetSy status groups (Table 1). However, white blood cell, neutrophil, and monocyte counts were significantly elevated in the MetSy group compared with the non‐MetSy group (*P* = .01, *P* = .02, and *P* = .04, respectively).

### Systemic Indices of the Immune System and Inflammation

Systemic indices of the immune system and inflammation were not significantly different between MetSy status groups (Table 2). Within the MetSy group, the systemic inflammatory indices did not show significant differences based on age and graft type (Table 3). However, NLR, PLR, SII, and PIV were significantly elevated in women compared with men (*P* < .01, *P* = .04, *P* < .001, and *P* < .01, respectively) (Table 3). The MLR and SIRI were not significantly different between women and men within the MetSy group. Additionally, NLR, MLR, PLR, SIRI, and SII were not significantly different based on obesity, dyslipidemia, low HDL, hypertension, and hyperglycemia criteria in the MetSy group. PIV was not significantly different regarding dyslipidemia, low HDL, hypertension, and hyperglycemia criteria, but it was significantly higher with BMI (≥30) (*P* = .02).

**TABLE 2:** Systemic Inflammatory Indices for MetSy Status and MetSy Criteria Components

	**NLR**	***P*** **Value**	**MLR**	***P*** **Value**	**PLR**	***P*** **Value**	**SIRI**	***P*** **Value**	**SII**	***P*** **Value**	**PIV**	***P*** **Value**
MetSy status		.28		.76		.13		.06		.42		.09
Yes (n = 50)	2.10 (1.10)		0.30 (0.10)		123 (55.9)		1.20 (1.00)		521 (313)		322 (284)	
No (n = 80)	1.90 (1.00)		0.30 (0.10)		134 (51.4)		1.00 (0.80)		507 (328)		251 (262)	
BMI		.79		.76		.61		.09		.13		.**01**
<30 (n = 56)	1.93 (1.09)		0.26 (0.11)		128 (47.0)		1.00 (0.80)		493 (335)		247 (228)	
≥30 (n = 74)	2.12 (0.95)		0.26 (0.11)		132 (58.0)		1.20 (0.84)		552 (352)		322 (289)	
Triglycerides		.72		.89		.07		.69		.34		.96
<150 mg/dL (n = 40)	1.99 (0.99)		0.26 (0.11)		134 (53.5)		1.05 (0.92)		549 (334)		263 (278)	
≥150 mg/dL (n = 90)	2.05 (0.99)		0.26 (0.13)		119 (52.0)		1.19 (0.90)		493 (293)		279 (256)	
HDL		**.04**		.72		.39		**.02**		**.04**		**.02**
Men ≥ 40 and women ≥ 50 mg/dL (n = 44)	1.93 (1.00)		0.27 (0.11)		134 (47.0)		0.98 (0.79)		501 (317)		250 (226)	
Men < 40 and women < 50 mg/dL (n = 86)	2.30 (1.04)		0.25 (0.12)		126 (63.5)		1.25 (1.04)		579 (408)		313 (411)	
Blood pressure		.96		.82		.82		.76		.69		.51
SBP < 130 and DBP < 85 mmHg (n = 71)	1.95 (1.09)		0.26 (0.11)		127 (48.3)		1.10 (0.91)		547 (384)		263 (277)	
SBP ≥ 130 or DBP ≥ 85 mmHg (n = 59)	2.05 (0.93)		0.26 (0.14)		134 (51.6)		1.09 (0.92)		507 (287)		271 (261)	
Estimated fasting glucose		.99		.33		.85		.11		.22		**.03**
<100 mg/dL (n = 87)	1.95 (0.95)		0.26 (0.10)		132 (42.3)		0.96 (0.73)		459 (283)		259 (220)	
≥100 mg/dL (n = 43)	2.04 (1.04)		0.27 (0.13)		127 (49.0)		1.15 (1.01)		551 (366)		294 (293)	

*Note*: Data are presented as median (IQR). Bold indicates *P* < .05.

BMI, body mass index; DBP, diastolic blood pressure; HDL, high‐density lipoprotein; IQR, interquartile range; MetSy, metabolic syndrome; MLR, monocyte‐to‐lymphocyte ratio; NLR, neutrophil‐to‐lymphocyte ratio; PIV, platelet‐to‐lymphocyte ratio; PLR, pan‐immune‐inflammation value; SBP, systolic blood pressure; SII, systemic immune‐inflammatory index; SIRI, systemic inflammation response index.

**TABLE 3:** Systemic Inflammatory Indices in the MetSy Group

	**NLR**	***P*** **Value**	**MLR**	***P*** **Value**	**PLR**	***P*** **Value**	**SIRI**	***P*** **Value**	**SII**	***P*** **Value**	**PIV**	***P*** **Value**
Sex		**<.01**		.75		**.04**		.06		**<.001**		**<.01**
Women (n = 26)	2.54 (1.02)		0.25 (0.12)		136 (48.1)		1.71 (0.98)		690 (500)		427 (455)	
Men (n = 24)	1.89 (0.57)		0.27 (0.13)		105 (60.0)		1.18 (0.55)		450 (173)		253 (126)	
Age		.52		.37		.38		.59		.37		.41
<40 years old (n = 30)	2.40 (1.15)		0.25 (0.14)		117 (63.0)		1.33 (0.95)		567 (334)		333 (318)	
≥40 years old (n = 20)	2.09 (0.73)		0.29 (0.11)		133 (50.1)		1.20 (0.68)		493 (260)		256 (238)	
Graft type		.85		.71		.98		.66		.88		.78
Allograft∗ (n = 23)	2.11 (1.01)		0.25 (0.11)		127 (55.5)		1.19 (0.52)		507 (331)		314 (196)	
Autograft† (n = 27)	2.33 (1.07)		0.27 (0.13)		120 (51.9)		1.38 (1.08)		554 (302)		329 (304)	
BMI		.80		.69		.50		.19		.08		**.02**
<30 (n = 10)	2.08 (0.79)		0.27 (0.08)		107 (33.2)		1.10 (0.41)		447 (197)		212 (150)	
≥30 (n = 40)	2.29 (1.19)		0.25 (0.13)		126 (59.0)		1.32 (0.99)		567 (361)		348 (372)	
Triglycerides		.72		.95		.89		.79		.72		.76
<150 mg/dL (n = 17)	2.29 (0.96)		0.25 (0.12)		108 (73.1)		1.19 (0.94)		551 (494)		330 (427)	
≥150 mg/dL (n = 33)	2.08 (1.15)		0.27 (1.12)		127 (42.2)		1.24 (0.88)		500 (285)		305 (249)	
HDL		.50		.07		.93		.90		.32		.59
Men ≥ 40 and women ≥ 50 mg/dL (n = 15)	1.91 (1.13)		0.32 (0.12)		106 (48.5)		1.27 (0.87)		500 (268)		329 (175)	
Men < 40 and women < 50 mg/dL (n = 35)	2.29 (0.99)		0.25 (0.10)		125 (58.3)		1.20 (0.98)		554 (342)		314 (372)	
Blood pressure		.32		.57		.91		.08		.17		.08
SBP < 130 and DBP < 85 mmHg (n = 10)	2.67 (0.97)		0.29 (0.07)		126 (25.6)		1.76 (0.65)		671 (337)		479 (202)	
SBP ≥ 130 or DBP ≥ 85 mmHg (n = 40)	2.09 (1.04)		0.25 (0.15)		118 (62.7)		1.16 (0.92)		503 (281)		287 (197)	
Estimated fasting glucose		.62		.72		.74		.85		.67		.85
<100 mg/dL (n = 4)	2.72 (0.52)		0.27 (0.17)		119 (36.7)		1.53 (0.42)		680 (164)		372 (110)	
≥100 mg/dL (n = 46)	2.11 (1.13)		0.26 (0.12)		123 (57.2)		1.20 (1.02)		510 (345)		310 (329)	

*Note*: Data are presented as median (IQR). Bold indicates *P* < .05.

BMI, body mass index; BTB, bone–patellar tendon–bone; DBP, diastolic blood pressure; HDL, high‐density lipoprotein; IQR, interquartile range; MetSy, metabolic syndrome; MLR, monocyte‐to‐lymphocyte ratio; NLR, neutrophil‐to‐lymphocyte ratio; PIV, pan‐immune‐inflammation value; PLR, platelet‐to‐lymphocyte ratio; SBP, systolic blood pressure; SII, systemic immune‐inflammatory index; SIRI, systemic inflammation response index.

∗ Allograft group includes hybrid graft (combination of autograft and allograft, n = 1).

† Autograft group includes BTB and hamstring autografts.

Within the non‐MetSy group, the systemic indices of the immune system and inflammation were not significantly different regarding sex, BMI, age, and graft type (Table 4). In addition, NLR, MLR, PLR, SIRI, SII, and PIV were not significantly different concerning BMI, low HDL, hypertension, and hyperglycemia criteria. NLR, MLR, and SIRI were not significantly different among triglyceride criteria; in contrast, PLR, SII, and PIV were significantly lower with dyslipidemia (*P* = .04, *P* = .01, and *P* = .04, respectively).

**TABLE 4:** Systemic Inflammatory Indices in the Non‐MetSy Group

	**NLR**	***P*** **Value**	**MLR**	***P*** **Value**	**PLR**	***P*** **Value**	**SIRI**	***P*** **Value**	**SII**	***P*** **Value**	**PIV**	***P*** **Value**
Sex		.33		.31		.65		.98		.23		.49
Women (n = 44)	1.99 (0.92)		0.25 (0.11)		134 (41.0)		0.92 (0.93)		556 (354)		262 (282)	
Men (n = 36)	1.92 (0.85)		0.27 (0.11)		130 (57.0)		1.06 (0.56)		465 (276)		245 (184)	
Age		.16		.38		.68		.08		.26		.25
<40 years old (n = 50)	1.97 (0.95)		0.26 (0.10)		136 (53.5)		1.06 (0.83)		536 (337)		262 (256)	
≥40 years old (n = 30)	1.92 (0.63)		0.25 (0.11)		129 (42.5)		0.84 (0.55)		463 (234)		209 (187)	
Graft type		.23		.12		.33		.57		.71		.68
Allograft∗ (n = 15)	2.05 (0.61)		0.30 (0.13)		144 (37.2)		1.01 (0.34)		558 (174)		263 (122)	
Autograft† (n = 65)	1.89 (1.04)		0.26 (0.10)		127 (47.3)		0.95 (0.83)		459 (350)		249 (282)	
BMI		.62		.96		.96		.79		.81		.44
<30 (n = 64)	1.92 (1.06)		0.26 (0.11)		130 (52.8)		0.98 (0.95)		508 (328)		249 (269)	
≥30 (n = 16)	1.99 (0.75)		0.26 (0.06)		136 (35.0)		0.97 (0.50)		492 (271)		262 (149)	
Triglycerides		.11		.52		**.04**		.17		**.01**		**.04**
<150 mg/dL (n = 73)	1.95 (0.91)		0.26 (0.11)		135 (50.4)		1.00 (0.91)		547 (331)		261 (268)	
≥150 mg/dL (n = 7)	1.54 (0.37)		0.21 (0.07)		91.5 (40.9)		0.72 (0.26)		375 (181)		183 (96.2)	
HDL		.09		.27		.98		.06		.09		.05
Men ≥ 40 and women ≥ 50 mg/dL (n = 71)	1.93 (0.91)		0.26 (0.10)		135 (48.2)		0.90 (0.70)		502 (320)		249 (244)	
Men < 40 and women < 50 mg/dL (n = 9)	2.53 (1.84)		0.27 (0.30)		132 (113)		1.39 (2.12)		658 (645)		311 (939)	
Blood pressure		.99		>.99		.62		.76		.94		.62
SBP < 130 and DBP < 85 mmHg (n = 49)	1.92 (0.95)		0.26 (0.11)		127 (52.6)		1.00 (0.70)		502 (341)		251 (256)	
SBP ≥ 130 or DBP ≥ 85 mmHg (n = 31)	1.95 (0.81)		0.26 (0.11)		135 (44.1)		1.60 (0.91)		511 (281)		249 (269)	
Estimated fasting glucose		.67		.45		.43		.37		.27		.08
<100 mg/dL (n = 39)	1.93 (0.89)		0.26 (0.09)		132 (41.1)		0.95 (0.66)		458 (242)		249 (148)	
≥100 mg/dL (n = 41)	1.93 (1.06)		0.27 (0.15)		135 (53.1)		1.00 (0.89)		566 (341)		274 (282)	

*Note*: Data are presented as median (IQR). Bold indicates *P* < .05.

BMI, body mass index; BTB, bone–patellar tendon–bone; DBP, diastolic blood pressure; HDL, high‐density lipoprotein; IQR, interquartile range; MetSy, metabolic syndrome; MLR, monocyte‐to‐lymphocyte ratio; NLR, neutrophil‐to‐lymphocyte ratio; PLR, platelet‐to‐lymphocyte ratio; PIV, pan‐immune‐inflammation value; SBP, systolic blood pressure; SII, systemic immune‐inflammatory index; SIRI, systemic inflammation response index.

∗ Allograft group includes hybrid graft (combination of autograft and allograft, n = 1).

† Allograft group includes BTB and hamstring grafts.

### MetSy Criteria Components and Systemic Indices of the Immune System and Inflammation

Independent of MetSy status, NLR, SIRI, and SII were not significantly different based on BMI, dyslipidemia, hypertension, and hyperglycemia criteria (see Table 2). However, NLR, SIRI, and SII were significantly elevated with low HDL (*P* = .04, *P* = .02, and *P* = .04, respectively). MLR and PLR were not significantly different based on individual MetSy criteria. PIV was not significantly different regarding the dyslipidemia and hypertension criteria. In contrast, PIV was significantly elevated with obesity, low HDL, and hyperglycemia (*P* = .01, *P* = .02, and *P* = .03, respectively).

### Postoperative Outcomes

Complication status, subsequent surgeries, reinjury status, and ED visits were not significantly different based on MetSy status (Figure 2). Patients who experienced postoperative complications exhibited significant elevations in MLR, SIRI, SII, and PIV (*P* = .03, *P* < .01, *P* = .03, and *P* < .01, respectively) (Figure 3). Conversely, the NLR and PLR were not significantly different based on reinjury status, subsequent surgeries, and ED visits. Finally, complication status, subsequent surgeries, reinjury status, and ED visits were not significantly different based on sex (Figure 4).

**FIGURE 2:**
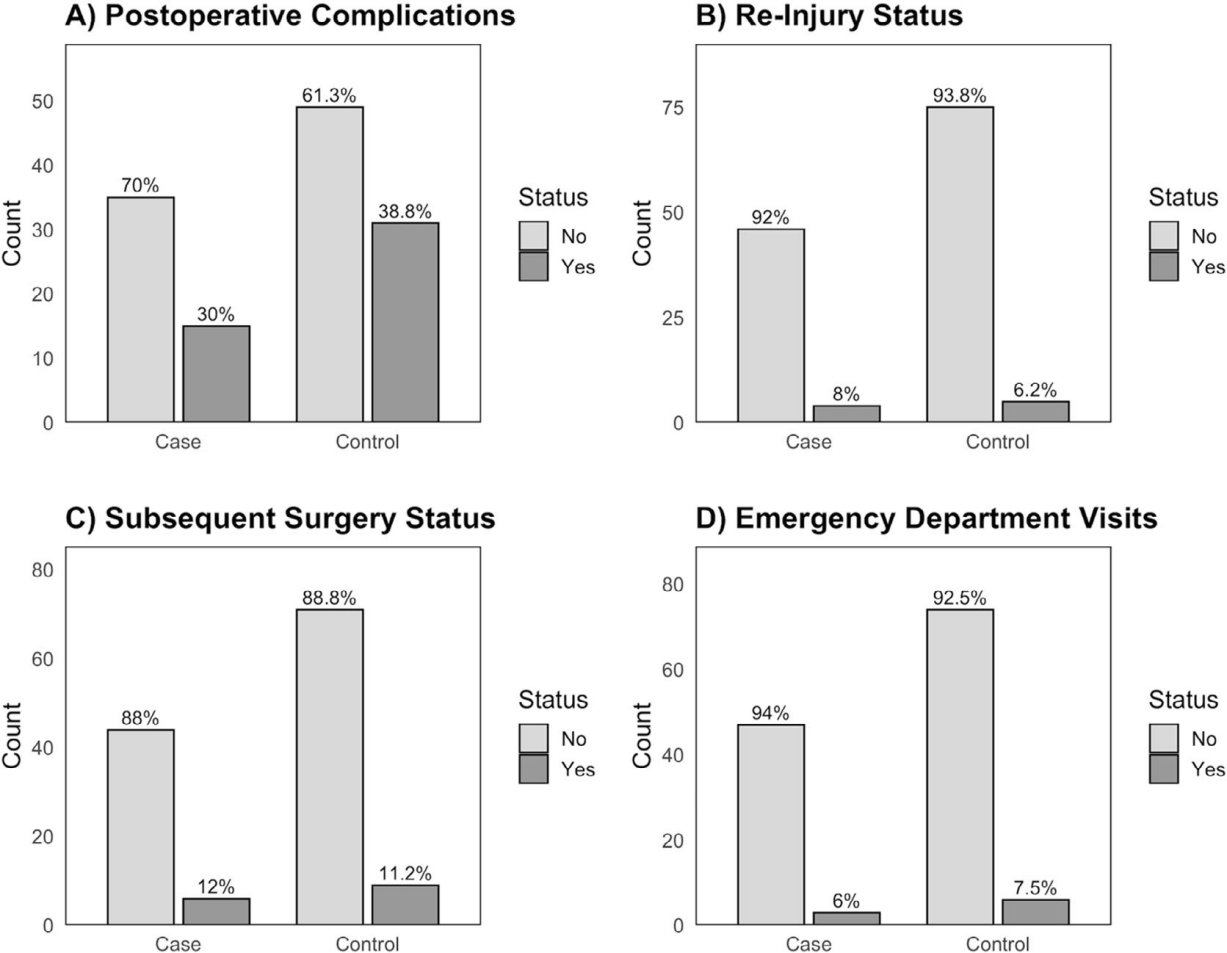
Postoperative outcomes and MetSy status. (A) Postoperative complication status, (B) reinjury status, (C) subsequent surgery status, and (D) emergency department visits were not significantly different between the MetSy group and non‐MetSy group (*P* = .41, *P* = .73, *P* > .99, and *P* > .99, respectively). Data are presented as count (%). (MetSy, metabolic syndrome.)

**FIGURE 3:**
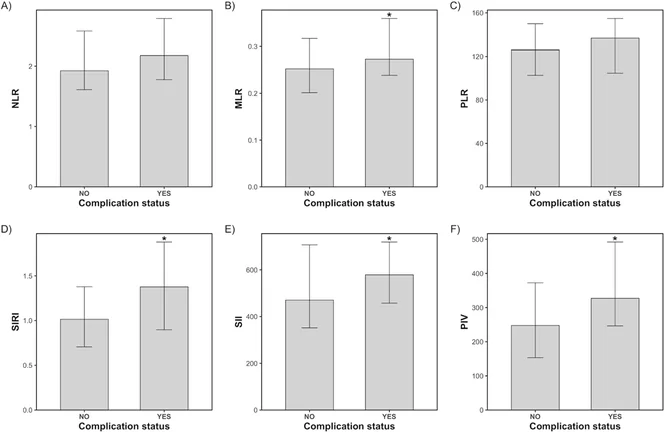
Systemic inflammatory indices and postoperative complication status. (A) NLR was not significantly different with postoperative complications compared with those without postoperative complications (*P* = .20). (B) MLR was significantly elevated with postoperative complications compared with those without postoperative complications (*P = .03). (C) PLR was not significantly different with postoperative complications compared with those without postoperative complications (*P* = .24). (D) SIRI, (E) SII, and (F) PIV were significantly elevated with postoperative complications compared with those without postoperative complications (*P < .01, *P = .03, and *P < .01 vs no complication status, respectively). Complication status was defined to include DVT, unexplained joint effusion, unexplained chronic pain, joint stiffness, quadriceps weakness, primary or progressive osteoarthritis, meniscus retear, limited ROM, arthrofibrosis, wound infection, cartilage degeneration, cyclops lesions, joint laxity, and recurrent knee instability. Data are presented as median (IQR). (DVT, deep vein thrombosis; IQR, interquartile range; MLR, monocyte‐to‐lymphocyte ratio; NLR, neutrophil‐to‐lymphocyte ratio; PIV, pan‐immune‐inflammation value; PLR, platelet‐to‐lymphocyte ratio; ROM, range of motion; SII, systemic immune‐inflammatory index; SIRI, systemic inflammation response index.)

**FIGURE 4:**
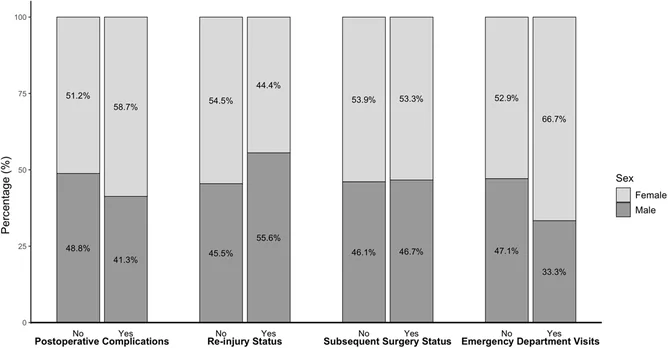
Postoperative outcomes between sexes. Postoperative complication status, reinjury status, subsequent surgery status, and emergency department visits were not significantly different between men and women (*P* = .46, *P* = .73, *P* > .99, and *P* = .50). Data are presented as percentages.

## DISCUSSION

The most important finding from this investigation indicates that the NLR, PLR, SII, and PIV were elevated in women with evidence of MetSy after ACLR compared with men. Although MetSy status alone was not associated with perturbed indices of the systemic immune system and inflammation, low HDL was associated with higher NLR, SIRI, SII, and PIV, whereas obesity and hyperglycemia were associated with higher PIV. These results highlight potential sex‐specific differences in systemic inflammatory markers after ACLR in patients with MetSy and its individual components. Although previous studies have revealed that substituting variables such as waist circumference and fasting glucose with alternative measures does not significantly alter ACLR outcomes,^21^, ^22^, ^24^ it is still possible that these substitutions may have introduced subtle or unmeasured inaccuracies in our dataset. As such, although the impact may be minimal, the potential for bias in determining MetSy cannot be entirely ruled out.

The present findings should be interpreted in the context of a prior publication from our laboratory,^25^ which evaluated systemic inflammatory markers after ACLR using the same institutional database. The prior study showed that older age at ACLR was associated with lower SIRI despite increased occurrence of a knee osteoarthritis diagnosis. In contrast, the current study found that MetSy alone was not associated with elevated indices of systemic inflammation, but rather specific MetSy components were associated with elevated systemic indices of inflammation. These findings collectively suggest that systemic inflammatory profiles may differ depending on whether patients are stratified by age or metabolic dysfunction, highlighting the multifactorial nature of immune and inflammatory responses in patients who underwent ACLR.

Another major finding was that specific systemic inflammatory markers—MLR, SIRI, SII, and PIV—were elevated in patients who experienced postoperative complications, independent of MetSy status or sex. These findings suggest that certain inflammatory indices can remain elevated postoperatively and correlate with higher complications, potentially used as a monitoring tool in future studies.

Previous studies suggest that higher levels of SII and PLR are positively associated with estradiol, whereas NLR is typically lower in women compared with men.^9^ Our findings partially align, showing higher levels of SII and PLR in women, along with elevated NLR and PIV. The reason for the discrepancy in NLR findings is unknown but could relate to sample characteristics because the results were limited to women with MetSy. CBC measurements were collected after injury and surgery, which may limit the generalizability.

Inflammation is essential to the early healing and recovery process, but an excessive or sustained inflammatory response can be detrimental to cellular and physiological functions. Previous research has linked systemic inflammation and MetSy to poor outcomes and mortality in elderly patients undergoing emergency surgery and in those with cardiovascular diseases.^1^, ^26^ Research on long‐term systemic inflammation in patients with MetSy after ACLR is scarce. Although MetSy is generally considered an inflammatory condition,^3^, ^10^, ^11^ our study indicates that whereas MetSy was not associated with systemic inflammation, individual MetSy components were linked to elevated indices. Limiting the present retrospective study to patients with available data in their electronic medical records potentially introduced selection bias by excluding patients without the prerequisite variables to determine MetSy. Another limitation of the present study was the lack of presurgery data and younger patient population, with a reported mean age of 37 years. It is also unknown if MetSy status and the presence of individual components were altered after ACLR. However, in agreement with previous studies, low HDL was correlated with higher SII,^11^ NLR, SIRI, and PIV, whereas hyperglycemia and obesity correlated with higher PIV. In contrast, the association between low HDL and inflammation was not observed within the MetSy group, which may be because of the multifactorial metabolic dysfunctional mechanisms that overshadow the independent contribution of HDL alone. Considering the heterogeneous complexity of MetSy and the prevalence of ACLR, further prospective research including pre‐ and postsurgery data is warranted.

In line with these findings, clinical outcomes after ACLR—including complication status, subsequent surgeries, reinjury, and ED visits—were not significantly different based on MetSy status. However, patients who experienced postoperative complications exhibited significantly higher MLR, SIRI, SII, and PIV. Moreover, complication status, subsequent surgeries, reinjury, and ED visits did not differ based on sex. These results suggest that certain systemic indices may be more predictive of postoperative complications than MetSy status or sex alone.

Although women exhibited higher systemic inflammatory indices after ACLR, this did not translate into higher rates of postoperative complications. This apparent discrepancy may reflect the multifactorial nature of surgical outcomes, where inflammation is only one of several contributing factors, including surgical technique, rehabilitation, and comorbidities. It is also possible that the elevations in systemic indices observed in women with MetSy, although statistically higher than men, did not exceed a threshold sufficient to increase postoperative complication risk. Additionally, protective biological or behavioral factors unique to women may have mitigated the impact of higher inflammation.

### Limitations

This study is not without limitations. First, the study size was small. The relatively small sample size may have limited the power to detect sex‐specific differences in complication rates, even though systemic indices were correlated with complications across the cohort. Second, the inclusion of patients with available laboratory data may introduce selection bias if testing was performed primarily for underlying (or suspected) medical conditions. It is worth noting that the study institution functions as a large integrated academic and primary care health system, where laboratory testing such as lipid panels and CBCs is frequently obtained as part of routine preventive care and annual physical examinations. Additionally, blood pressure measurements used in defining MetSy are routinely recorded at clinical visits. Therefore, laboratory availability likely reflects both routine health maintenance and clinical evaluation rather than selective testing alone.

## CONCLUSIONS

Indices of systemic inflammation after ACLR were elevated in women with evidence of MetSy. Patients with postoperative complications also exhibited an elevation in systemic indices of inflammation after ACLR.

## DISCLOSURES

The authors (M.A., S.B., H.P., H.A.J., C.R., A.M., N.A., C.C.K., R.A.M., D.C.F., T.B.) declare that they have no known competing financial interests or personal relationships that could have appeared to influence the work reported in this article.
